# Stem-Pessary

**Published:** 1877-10

**Authors:** 


Dr. Taliaferro, of Atlanta, publishes in the Richmond and Louisville Magazine for August a long article upon stem-pessaries, wherein the subject is quite well set forth. The occasion for the paper was a criticism which appeared in the Louisville Medical News (May 26th) on Dr. Taliaferro's stem-pessary. It may be remembered that the peculiarity of this instrument was its archaic simplicity, and one being able to fashion it, and the fact that it was to be stitched *in situ.* We admire Da. Taliaferro's industry, as displayed in the list of authorities presented on the main subject of stem-pessaries, and we acknowledge the courtesy shown us in not muffling *our* heads with the mantle of his contempt. We might have wished that he had said more concerning his own invention, which was the only point at issue, simply that we might transcribe the same for the benefit of our readers. Personally, the wood-cut given of the Taliaferro stem-pessary is amply satisfactory. We can see at a glance now how unjust was the criticism, as this shows Dr. Taliaferro simply wishes to insert a concern which looks like the Bunker Hill Monument into the cervical canal, and hold it there with a few guy-ropes passed through the uterine walls. We had feared he intended to be rough.—*Louisville Medical News.*

